# Culture, communication and community in palliative cancer care: a view from India

**DOI:** 10.3332/ecancer.2022.ed120

**Published:** 2022-04-28

**Authors:** Dwaipayan Banerjee

**Affiliations:** Science, Technology, Society, Massachusetts Institute of Technology, E51-194A, 77 Massachusetts Avenue, Cambridge, MA 02139, USA

**Keywords:** palliative care, India, disclosure, cancer, community health

## Abstract

Two palliative cancer-care models are being pioneered in India. The first has been developed by CanSupport, a cancer-care NGO based in Delhi. The CanSupport model of care emphasizes expertise and focuses on a relatively small number of patients. The second model is the Neighbourhood Network of Palliative Care, advanced by a group of physicians in the southern state of Kerala. The NNPC model emphasizes reach over expertise. It provides short-term training to community workers and civic-minded citizens, mobilizing numbers to treat a wider patient pool. This paper compares the strengths and drawbacks of both models in order to understand the generalizability of each for providing care to lower-income communities in lower- and middle-income countries.

This work was presented at the Palliative Care, Culture and the Clinic symposium in January 2021 and the accompanying Special Issue from the event can be found here: https://ecancer.org/en/journal/special-issue/32-biomedicine-and-the-soul-in-relation-to-global-palliative-care.

## Background

A 2018 special report commissioned by the Lancet found that in 2015 alone, about twenty million people in lower- and middle-income countries died with serious pain and most of them without access to pain relief. Other reports confirming the seriousness of this problem find India at the centre of the global pain epidemic. For example, the American Cancer Society report found that about 24 percent of worldwide deaths accompanied by end-of-life pain happened in India alone. In response to this pain epidemic, several organizations (both state-led and non-governmental) have mobilized efforts to provide palliative cancer care. In doing so, some emphasize reaching the greatest number of people while others argue for the need to deliver specialized care without diluting the expertise required for effective palliative care work.

## Methods

The research in this paper is based on ethnographic work conducted in 2011-2012 with CanSupport - Delhi’s largest palliative cancer-care NGO. I followed about ten of the NGO’s teams as they provided home-based care to patients. Over my time with the organization, I was able to visit the homes of about a hundred patients. More than half of these patients were among the urban poor who lived in formal and informal settlements. In addition, I observed palliative care work at the cancer center of Delhi’s leading public hospital and conducted an exhaustive survey of the research literature on palliative care in India.

## Discussion

In 2005, a heated debate broke out in India’s flagship pain and palliative care forum, the Indian Journal of Palliative Care. On one side of the debate were palliative care practitioners from the state of Kerala in southern India. On the other were practitioners from Delhi. Palliative cancer care in Kerala has taken a community-oriented form, led by a coalition of four organizations under the rubric of the Neighbourhood Network of Palliative Care (NNPC). They argued that palliative care should be understood as an integral part of primary health care. Further, they argued that “emic volunteers” within neighbourhoods were best attuned to the needs of patients. The essence for their argument was that community ownership of health care would lead to better health outcomes for the broadest overall patient population.

Others, however, questioned the generalizability of the Kerala model. For instance, Harmala Gupta, the founder of CanSupport astutely pointed to the NNPC’s idealization of the concepts of “communities” and “participation”.

There is a tendency amongst us to mourn the loss of a traditional past with its sense of a closely knit and concerned community. Yet, when we look closely at the requirements of palliative care delivery, can we overlook the specifics of the dying patient’s deepest needs? Are the interests of this sick person best served by amorphous interventions extended by well-meaning people, perhaps even neighbors, or by trained professionals comprising doctors, nurses, and counselors? [1]

Thus, Gupta suggested that such an expansion of the mode of treatment would lead to a dilution in the quality of care and a dangerously thin understanding of the social context in which palliative care treatment was delivered.

A primary aspect of the social context at stake is the secrecy and nondisclosures of cancer diagnosis. For example, early in my fieldwork I was struck by how rarely the word cancer was ever spoken. In one instance, I travelled with CanSupport to a patient who lived in the outskirts of the city. Our car had broken down, and we travelled the last miles in the official NGO van with its logo “Caring for Cancer” printed on the door. The patient— Amarjit (pseudonym)—seemed visibly discomfited by the logo: he absolutely did not have cancer, he said. In his refusal to name his diagnosis, he was exemplary of many others who resisted enclosing the disease within an already fixed script. The nurse expertly played along, hoping to transact care on his terms rather than her own. She asked, “What do you think has happened to you?)” His careful reply was that he had “oncology,” a careful negotiation of the word “cancer” and all that the diagnosis entailed. It was why CanSupport almost always entered neighbourhoods as discretely as possible, in a bid to respect the strategies of those under their care.

This tricky relationship between concealment and disclosure appeared consistently throughout my fieldwork as a fundamental aspect of the problem with delivering effective palliative care in Delhi. Looking through more than six hundred patient records at the cancer centre of North India’s largest public hospital, I found that more than 80 percent had been recorded as being “unaware” of their diagnosis when they came to the clinic. Patients and families often came to the ward and hid prognoses they had received from other doctors or oncologists, believing that revealing a bleak prognosis would hurt their chances of accessing care. In this, they revealed an underlying lack of trust between patients and doctors. At other times, patients and families colluded to conceal their diagnosis from neighbours and kin. And most frequently, family members colluded with each other to protect patients from the perceived psychic impact of the word. Thus, the patient was really never ‘unaware’ of their own diagnosis; *concealment was part of a set of strategies to manage the impact of diagnosis.*

Importantly, in the public health literature, concealment is taken as evidence of non-compliance and a way to evade treatments. As Alex Broom and Assa Doron show, many cancer physicians in India too understand nondisclosure as indicative of ignorance and denial, even as they participate and collude in the act [2]. Yet, I found nondisclosures to evidence neither ignorance nor denial, but rather a way through which patients negotiated the burdens of the disease, especially when neighbours and kin were not reliable sources of support. Concealing diagnoses rarely detracted from seeking appropriate biomedical care. Rather, the biggest barrier to healthcare remained an over-stressed public health system not geared towards treating the disease at scale.

What does this debate between two most prominent cancer-care organizations tell us about the possibilities of providing effective palliative care, and especially to lower-income patients? Understanding their very different origins helps to answer this question. The WHO formalized the framework of community participation as one of its guiding principles in 1978 [3, 4]. It remains a guiding principle in many global health policy proposals such as the UN Millennium Development Goals and the World Bank’s poverty reduction strategy. While the model was unevenly implemented elsewhere in the country, it took deep roots in Kerala. In 1996, the newly elected Left Democratic Front Government in the state launched an ambitious plan to decentralize planning and promote community participation in government [5]. This devolution of power was a response to years of activism, civil society mobilization and a slow change in vision within the Communist Party of India (CPI-M).

At present, Kerala far surpasses the rest of the country according to most indicators of healthcare quality [6, 7]. While it only has three percent of the country’s population, a study published in 2008 found that of the 139 points of delivery for palliative care in the country, 83 were based in Kerala [8]. In 2008 the government consulted with the state’s leading palliative care professionals to draft an official state policy regarding the practice. It recognized the NGOs engaged in the work and allocated significant resources to help them extend the model for the entire state. It is no surprise then that the success of the Kerala model has attracted international attention, leading to many healthcare partnerships between organizations such as the WHO and the government of Kerala

In stark contrast, the Delhi government took sixteen years to respond to court directives to ease its drug control policies and make oral morphine available to cancer patients. In the meantime, it had taken CanSupport five years to procure their license to distribute morphine. Delhi is very different from Kerala then in that it has not seen the same size and scale of historical mobilizations for equal access to healthcare. It is in this very different context that the CanSupport position makes sense. Confronted with a limited healthcare system and without the direct support of the government, CanSupport has to raise its own funds and therefore the scale of its interventions is smaller. Equally importantly, the absence of adequate healthcare is one of the factors contributing to the secrecy and stigma around the diagnosis. Cancer remains strongly associated with death, and so neighbors and kin often withdraw rather than intervene to provide significant support.

## Conclusion

Understood in this way, neither model is right or wrong. Each is an outcome of their vastly different socio-political contexts. With adequate state support, the two can go hand in hand, with expert-driven teams working alongside a civic-minded population mobilized to demand and expect access to healthcare, including palliative interventions.

## Conflicts of interest

The author declares no conflict of interest.

## Funding

This research was funded by grants from the National Science Foundation and the Wenner Gren foundation.
